# Activity‐Based Functional Exercise for Disability in Systemic Sclerosis: Protocol for a Feasibility Randomised Controlled Trial

**DOI:** 10.1002/msc.70217

**Published:** 2026-03-30

**Authors:** Gabriela da Silva Santos, André Nunes Soares, Geisielly Maria Porto Soares, Thaís Ariadne Medeiros de Lima, Juliana Fernandes, Angela Luzia Branco Pinto Duarte, Angélica da Silva Tenório, Andrea Tavares Dantas

**Affiliations:** ^1^ Department of Physical Therapy Federal University of Pernambuco Recife Brazil; ^2^ Department of Clinical Medicine Federal University of Pernambuco Recife Brazil

**Keywords:** exercise, feasibility studies, functional status, physical therapy, quality of life, systemic sclerosis

## Abstract

**Introduction:**

Systemic Sclerosis (SSc) is associated with substantial disability in affected patients. Although exercise interventions for SSc have been developed, most focus on isolated body segments rather than global disability. Thus, this study aims to assess the feasibility of a functional activity‐based exercise programme designed to address disability in individuals with SSc.

**Methods:**

This feasibility study protocol is designed as a randomised controlled mixed‐methods trial. It will include individuals with SSc aged 18–65 years and with a score ≥ 1 on the Scleroderma Health Assessment Questionnaire (SHAQ). Participants will be randomly allocated to either a control group, receiving an educational booklet on the disease, or an intervention group receiving the booklet plus a 10‐week supervised functional exercise protocol (twice weekly). Feasibility outcomes include recruitment and retention rates, adherence, safety, and adverse events. Preliminary clinical outcomes will address disability level, physical function, fatigue, resilience, symptoms of anxiety and depression, and perceived change. Quantitative data will be analysed using descriptive and inferential statistics, while qualitative data will undergo thematic content analysis.

**Ethical Approval:**

The study was approved by the Institutional Ethics Committee (Approval Number: 6.992.710).

**Results:**

The study is in its initial phase. The reference clinic has 219 registered patients, of whom 183 (83.6%) are preliminarily eligible. Recruitment and subsequent feasibility results are ongoing.

**Conclusion:**

This protocol proposes an innovative and low‐cost approach, whose results will support refinements for a more rigorous clinical trial, despite potential logistical and disease‐related limitations.

## 1 Introduction

Systemic sclerosis (SSc) is a chronic multisystem disease that is associated with significant disability (Zaghlol et al. 2022). The International Classification of Functioning, Disability and Health (ICF) defines disability as the difficulty an individual experiences in performing daily activities or participating in society (Organização Mundial da Saúde 2008). In SSc, disability can be attributed to the cumulative impact of multiple impairments caused by or associated with the disease (Jaeger et al. 2018; Khanna et al. 2017; Papelard et al. 2019; Yetisir et al. 2025).

The management of SSc is based on continuous assessment to address potential complications, with the implementation of both pharmacological and non‐pharmacological strategies (Thoreau et al. 2021). Patient education and physical exercise are the most studied non‐pharmacological approaches. However, most exercise protocols focus on specific body segments, such as hand function (Bongi et al. 2011; Piga et al. 2014; Santos Cardozo Roque et al. 2022).

The ICF *core set* developed for SSc patients identified significant limitations in global functions within the *activity and participation* domain. These include daily routines, postural changes, object manipulation, walking, household tasks, and leisure activities (Papelard et al. 2019). These findings reinforce the need for exercise therapies that address more than isolated body segments.

Thus, we hypothesise that a functional activity–based exercise programme reduces the disability in individuals with SSc, thereby improving perceived quality of life and reducing the disease's impact on patients' lives. Accordingly, the objective of the future randomised controlled trial (RCT) will be to evaluate the effect of a functional activity–based exercise programme on functionality in individuals with SSc compared to a control group.

However, prior to conducting a RCT, a feasibility study will be performed to assess the viability of the proposed methodology and intervention protocol. This step allows for necessary adjustments before proceeding with the full trial. Therefore, the objective of this study is to assess feasibility‐related outcomes, including recruitment, retention, the acceptability, adherence and safety of the intervention, as well as to report on intervention costs, and the occurrence of any adverse events.

## 2 Methods

### 2.1 Design and Setting

This study protocol describes a mixed‐methods randomised controlled feasibility study. To be conducted at the rheumatology outpatient clinic of the Hospital das Clínicas at the Federal University of Pernambuco (HC‐UFPE) (a reference centre for SSc care in the state of Pernambuco) and at the Laboratório de Aprendizagem e Controle Motor (LACOM).

### 2.2 Participants

The study includes individuals aged 18–65 years, of any sex, with a score ≥ 1 on the *Scleroderma Health Assessment Questionnaire* and a diagnosis of SSc based on the 2013 classification criteria from the American College of Rheumatology/European League Against Rheumatism (van den Hoogen et al. 2013).

Exclusion criteria are: pregnancy; active malignancy; recent (3 months) changes in pharmacotherapy; physical therapy treatment in the past 3 months; overlap with other autoimmune rheumatic diseases that primarily affect the musculoskeletal system; presence of conditions that limit physical activity (e.g., severe neurological impairment, immobility or advanced cardiopulmonary symptoms); individuals who engage in regular physical exercise (at least three times per week); cardiac symptoms classified as class III or IV according to the New York Heart Association (Caraballo et al. 2019); and joint surgery within 6 months prior to study inclusion. Eligibility was determined through clinical assessment.

### 2.3 Procedure: Screening, Informed Consent, Randomisation, Allocation Concealment, and Blinding

The researcher will recruit patients directly in the HC‐UFPE waiting room. When inviting participation, the study procedures are explained, and if interest is shown, a preliminary eligibility check is conducted based on self‐reported information and clinical records to verify inclusion and exclusion criteria. The reasons for including or excluding each individual are recorded.

If the individual is eligible and agrees to participate, they receive the Informed Consent Form (ICF) for review and signature. No research‐related assessments, data collection, or intervention procedures were initiated prior to the signing of the informed consent.

After consent is obtained, participants are randomised into one of the two study groups. Randomisation uses block allocation (*n* = 10) via sealed opaque envelopes. Envelopes are opened only after the participant's baseline assessment.

Data collection was performed by Examiner A or B, both of whom were blinded to group allocation. Participants are instructed not to disclose to researchers A and B the group to which they were allocated. The exercise protocol was administered by Examiner C, who will be the only one aware of the participants' group assignments.

### 2.4 Study Groups

Control group (CG): Participants receive an educational booklet on SSc developed by the Brazilian Society of Rheumatology (Sociedade Brasileira de Reumatologia and Comissão de Esclerose Sistêmica 2025). For those with reading difficulties, a research member will provide a video explanation via WhatsApp. Additionally, participants are instructed not to begin any physiotherapeutic intervention or exercise practice that could affect their physical conditioning during the study. After the final assessment, participants in this group will be offered follow‐up care using the study's intervention protocol.

Functional exercise group (FEG): In addition to receiving the same booklet (or video) as the CG, participants in this group individually perform a supervised functional activity‐based exercise protocol led by a physiotherapist/researcher.

### 2.5 Intervention

The protocol was developed by experienced physiotherapists specialised in the care of patients with rheumatic diseases, with consultation from a medical specialist in SSc. It is structured based on movements commonly used in activities of daily living (Papelard et al. 2019). The intervention lasts 10 weeks, with individual sessions held twice a week at the Physiotherapy Clinic of UFPE.

Each session is divided into three phases:

1. Mobility phase—includes range‐of‐motion exercises (diagonal trunk flexion, wrist rotation, hip and knee flexion and trunk rotation) to begin the session gently. These exercises are carried out in the same manner throughout the entire intervention period.
2. Warm‐up phase—includes upper limb cycling for 2 minutes and lower limb cycling for 4 minutes, performed throughout the entire intervention period.
3. Functional activity‐based exercises—include pelvic lifts, upper limb mobility, trunk diagonals, sit‐to‐stand, step‐ups and step‐downs, walking circuits, and hand strengthening and mobility exercises. These exercises are divided into movements with progressive levels of complexity and load, thus aiming to approximate the training conditions to daily life activities.

Exercise difficulty progresses over the course of the intervention. The first week focuses on introducing the movements. From the second week onward, the protocol is organised into three blocks of 3 weeks each, with exercises progressing gradually within each block.

The full protocol is available in the Supporting Information S1 of this article.

## 3 Outcomes and Measures

### 3.1 Feasibility Assessment

To assess the feasibility of the study, the following data will be collected (Pearson et al. 2020):

1. Eligibility rate: the percentage of patients registered at the outpatient clinic who were eligible for recruitment;
2. Recruitment rate: the proportion of eligible individuals who agree to participate. The number of participants recruited per month will also be estimated;
3. Randomisation rate: the proportion of recruited participants who were successfully randomised;
4. Retention rate: the proportion of participants who completed all planned assessments. A rate of 70% will be considered adequate (Teresi et al. 2022);
5. Adherence rate: the proportion of participants who completed the entire protocol;
6. Protocol fidelity: documented by recording any unplanned adaptations, including their reasons and types;
7. Acceptability: assessed through participants' self‐reported difficulties or discomfort related to the protocol and the study. These reports will be collected as notes during each session. Acceptability will also be explored through qualitative data from semi‐structured interviews, focusing on participants' opinions and challenges during protocol implementation.

### 3.2 Study Measures

Disability level is assessed using the Scleroderma Health Assessment Questionnaire (SHAQ) (Pope 2011), in which higher scores indicate greater disability. SHAQ is validated for Brazilian Portuguese (Rocha et al. 2014). In addition, lower limb physical performance is evaluated through the 30‐s sit‐to‐stand test (30sSTS) (de Santana et al. 2014) and the 6‐min walk test (6MWT), a valid and reproducible measure of functional capacity in patients with systemic sclerosis (Pugnet et al. 2018); and handgrip strength is assessed using manual dynamometry with a Jamar dynamometer, following the procedures described by Mathiowetz et al. 1984; (Mathiowetz et al. 1984).

The following outcomes were assessed using questionnaires validated for Brazilian Portuguese, with adequate psychometric properties: fatigue severity using the Fatigue Severity Scale (FSS) (Krupp et al. 1989; Mendes et al. 2008); upper limb disability using the Cochin Hand Functional Scale (CHFS) (Chiari et al. 2011; Poole 2011); symptoms of anxiety and depression using the Hospital Anxiety and Depression Scale (HADS) (Botega et al. 1995; Zigmond and Snaith 1983); resilience using the Connor‐Davidson Resilience Scale (CD‐RISC‐10) (Campbell‐Sills and Stein 2007; Solano et al. 2016); and perceived changes following the intervention using the Patient Global Impression of Change (PGIC) (Domingues and Cruz 2011; Guy and National Institute of Mental Health, Psychopharmacology Research Branch and Early Clinical Drug Evaluation Program 1979).

### 3.3 Data Collection Procedures

Table 1 summarises the study phases and procedures. First, sociodemographic and clinical data are collected. Then, data related to the study outcomes are obtained.

**TABLE 1:** Study procedures.

Procedures	Enrolment	Post‐randomisation	Intervention (Weeks 1–10)	Close‐out
Enrolment				
Eligibility screening	X			
Informed consent	X			
Randomisation		X		
Intervention or control				
Intervention^a^			X	
Control^b^			X	
Assessment				
Disability‐related questionnaires		X		X
Physical tests		X		X
Semi‐structured interview		X		X

^a^ Functional activity‐based exercise protocol administered twice weekly for 10 weeks.

^b^ Usual care.

After that, the participant takes part in a semi‐structured interview. They are informed that the audio is recorded solely for transcription purposes and that their identity is protected at all times. Examiner A conducts the interview using a predefined script. However, it encourages the participant to speak freely, with no restrictions on time or content, ensuring that all opinions and reflections are thoroughly recorded. The interview is divided into two phases: a pre‐intervention component conducted at baseline to assess participants' expectations regarding the intervention, and a post‐intervention component conducted after completion of the protocol to explore participants' perceptions of the intervention.

### 3.4 Sample Size

Because the purpose of this study was to assess feasibility rather than the effectiveness of an intervention, no formal sample size calculation was performed. However, a recruitment target of 12 participants per group was established, as previously recommended (Julious 2005).

### 3.5 Data Storage and Sharing

Printed data are stored in physical files, while audio recordings and transcripts are saved digitally at the Federal University of Pernambuco. All data are retained by the principal investigator for at least 5 years.

Data sharing may occur upon a justified request to the corresponding author.

### 3.6 Data Analysis

Data will be analysed using SPSS version 22.0, adopting a significance level of 5% (*p* < 0.05). All analyses will be conducted according to the intention‐to‐treat principle, with participants analysed in the groups to which they were originally randomised, regardless of adherence to the intervention protocol. As this is a feasibility study, analyses are not intended to estimate definitive treatment effects. Protocol adherence will be reported descriptively as a feasibility outcome.

Normality will be assessed using the Shapiro–Wilk test. For baseline characteristics and pre‐intervention outcomes, quantitative variables with normal distribution will be compared using the independent Student's *t*‐test. Nonparametric distributions will be analysed using the Mann–Whitney test. Categorical variables will be compared using Yates' correction, Fisher's exact test, or Pearson's chi‐square test, as appropriate.

Post‐treatment outcomes will be evaluated using analysis of covariance (ANCOVA). Each post‐intervention outcome will be modelled as a function of group (categorical factor) and its corresponding baseline value (linear covariate). Results will be expressed as mean ± standard error.

Effect size will be calculated using Cohen's d, and interpreted as follows: < 0.2 as negligible; ≥ 0.2 to ≤ 0.5 as small; ≥ 0.5 to ≤ 0.8 as moderate; and > 0.8 as large (Gignac and Szodorai 2016).

For qualitative data, the transcribed interviews will be subjected to thematic content analysis according to the Bardin framework (Bardin 2011).

### 3.7 Adverse Events

Participants will be monitored for adverse events during each session. All reported or observed events will be recorded in monitoring forms by the responsible researcher.

### 3.8 Ethical Approval

This study was conducted in full compliance with the ethical principles outlined in Resolution 466/12 of Brazil's National Health Council and the Declaration of Helsinki. Ethical approval was granted by the Institutional Ethics Committee under approval number 6.992.710 and CAAE registration 81341724.2.0000.5208.

## 4 Results

The intervention protocol was registered with the Brazilian Registry of Clinical Trials (ReBEC) under the code RBR‐10nbcsbw. The study is currently in its initial phase.

In collaboration with healthcare professionals from the rheumatology outpatient clinic at HC‐UFPE, a survey was conducted to determine the total number of patients registered as receiving care. The number of patients preliminarily eligible for research participation was then assessed. The outpatient clinic currently has 219 patients under care. Of these, 183 are eligible for the present research.

The next phase of the research will involve the initiation of screening procedures. Screening will be conducted weekly on Wednesdays, when the outpatient clinic provides care for patients with SSc. On average, 11 patients are scheduled per day. The principal researcher will approach potential participants in the waiting room.

## 5 Discussion

This protocol could serve as a low‐cost, easily adoptable option for physiotherapists, requiring no additional training for implementation. Its innovativeness is also significant. Although previous studies have investigated physical exercise in patients with SSc (Melchiorre et al. 2023; Parodis et al. 2023; Santos Cardozo Roque et al. 2022), most do not evaluate global disability as a primary outcome. To the authors' knowledge, no similar intervention has been proposed to date.

Potential limitations include logistical challenges related to participant attendance, a small sample size, and disease‐related complications that may influence adherence or outcomes independently of the intervention. In addition, the intervention was not developed with direct patient involvement. However, the collection of qualitative data on participants' experiences and feedback will provide valuable insights to guide future refinement of the protocol.

Uncertainty regarding the sensitivity to change of the selected outcome measures in systemic sclerosis is another limitation. Nonetheless, examining the behaviour and variability of these outcomes aligns with the objectives of a feasibility study and will inform the design of subsequent efficacy trials.

We anticipate that the study results will be published in peer‐reviewed journals, presented at regional and national conferences, and shared with participants who wish to receive this information.

## Author Contributions

Gabriela da Silva Santos contributed to conceptualization, data curation, investigation, methodology, project administration, visualization, and writing of the original draft, as well as review and editing. Andrea Tavares Dantas contributed to conceptualization, data curation, methodology, project administration, supervision, visualization, and review and editing. Angélica da Silva Tenório contributed to conceptualization, methodology, and supervision. Juliana Fernandes contributed to methodology, supervision, and review and editing. André Nunes Soares, Geisielly Maria Porto Soares, and Thaís Ariadne Medeiros de Lima contributed to investigation and data analysis. Angela Luzia Branco Pinto Duarte contributed to validation and review.

## Funding

The Article Processing Charge for the publication of this research was funded by the Coordenação de Aperfeiçoamento de Pessoal de Nível Superior ‐ Brasil (CAPES) (ROR identifier: 00x0ma614).

## Conflicts of Interest

The authors declare no conflicts of interest.

## Supporting information


Supporting Information S1

## Data Availability

The data that support the findings of this study are available from the corresponding author upon reasonable request.
