# Supplementary material for: Activity‐Based Functional Exercise for Disability in Systemic Sclerosis: Protocol for a Feasibility Randomised Controlled Trial
Source: Musculoskeletal Care. 2026 Mar 30;24(2):e70217. doi: 10.1002/msc.70217 (PMC13035445; doi:10.1002/msc.70217)
Supplement: Supplementary file 1 — Supporting Information S1 [file MSC-24-e70217-s001.pdf]

Table 1. Exercises and progressions of the intervention protocol					
**Category**	**Exercise**	**Week 1**	**Weeks 2–4**	**Weeks 5–7**	**Weeks 8–10**
**Mobility**	**Diagonal trunk flexion**	Seated, lean the trunk forward diagonally as if trying to reach one foot. 3 repetitions per side.			
	**Wrist rotation**	Seated, arms extended forward, perform circular wrist movements. 3 repetitions (clockwise and counterclockwise).			
	**Hip and knee flexion**	Standing (with optional backrest support), flex one leg at the hip, knee, and ankle. 3 repetitions per leg.			
	**Trunk rotation**	Standing, upper limbs extended forward, rotate trunk while opening one arm to the side (alternating sides). 3 repetitions per side.			
**Warm-up**	**Upper and lower limb cycling**	2 minutes of upper limb cycling on cycle ergometer and 4 minutes of lower limb cycling on stationary bike.			
**Bridge**	**Pelvic lift**	Combined with scapular adduction and shoulder flexion (holding a ball with	Combined with hip abduction (miniband on knees), scapular adduction, and shoulder flexion	Combined with scapular adduction and shoulder flexion (miniband on forearms) and bilateral	Combined scapular adduction and shoulder flexion (with miniband on the forearms) and bilateral pelvic rotation*. In

		extended elbows) – 8 reps	(miniband on forearms) – 8–10 reps	pelvic rotation* – 8–10 reps	addition, in the movement of raising and lowering the pelvis, isometric contraction will be performed for 5 seconds - 8 to 10 repetitions
**Diagonal trunk**	**Diagonal trunk flexion**	Therapist throws ball in various directions to stimulate trunk rotation – 10 reps	Seated diagonal trunk flexion to transfer object (predefined load) between points: shoulder height → knee height - 8-10 repetitions	Standing diagonal trunk flexion to transfer object (predefined load) between points: shoulder height → knee height - 8-10 repetitions	Standing: transfers object (predefined load) between points - head height → mid-leg level - 10 repetitions
**Sit-to-Stand**	**Sit-to-stand from a chair**	Partially assisted: Hands on thighs,	Semi-assisted: Partial stand with free hands	Unassisted: Full sit-to-stand with free hands – 8–10 reps	With control: Full sit/stand with isometric pauses (5s

		partial stand (3s isometric) – 8 reps	(5s isometric) – 8–10 reps		mid-movement) – 8–10 reps
**Step training**	**Step/ stair ascent and descent ****	Single step with control: Pause (3s mid-movement) – 8 reps per leg	Three continuous steps: ascend/descend without load – 8–10 reps	Light load: 3-step climb/descend holding 0.5 kg bags – 8–10 reps	Progressive load: 3-step climb with 1.0 kg bags – 8–10 reps
**Gait**	**Gait circuits**	Basic: Seated → stand → walk to cone → return (5 laps)	Intermediate: Seated → stand → step up/down (15 cm) → 2 cones → return (5 laps in week 2; 8 after)	Complex: Seated → stand → step → 2 obstacles → 3 cones → return (5 laps week 5; 8 after)	With load: Seated → stand (carrying 1kg bag/hand) → step → 2 obstacles → 3 cones → return (8 laps week 8; 10 after)
**Hand exercises**	**Mobility and strengthening**	Basic mobility:<br>- Finger flexion/extension (10x/hand)	Coordination + initial resistance:<br>- Transfer 10 varied pieces between	Moderate resistance:<br>- Transfer 10 pieces per hand<br>- Open/close fingers	Advanced resistance:<br>- Transfer 20 pieces between containers<br>- Open/close fingers with

		- Finger abduction/adduction (10x/hand)<br>- Roll/unroll cylindrical object (8x)	containers (1x/hand)<br>- Open/close fingers against weak theraband (8x/hand)<br>- Roll/unroll cylindrical object (5x each way)	with moderate theraband (10x/hand)<br>- Roll/unroll cylindrical object (10x each way)	moderate theraband (10–12x/hand)<br>- Roll/unroll cylindrical object (12x each way)
* The individual performs the bridge exercise, then returns and moves the lower limbs (knees flexed and feet supported) in pelvic rotation to one side, then the other; repeating both the bridge and rotation as prescribed.<br>** Steps may include optional handrails.					

Below are images illustrating each of the exercises and their progress over the weeks.

MOBILITY

Diagonal trunk flexion

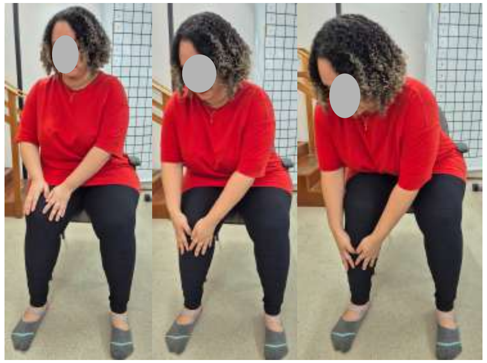

Wrist rotation

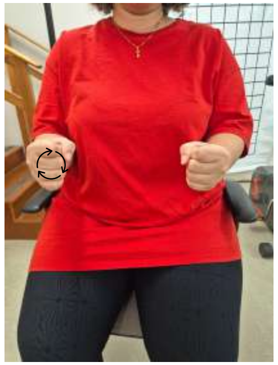

Hip and knee flexion

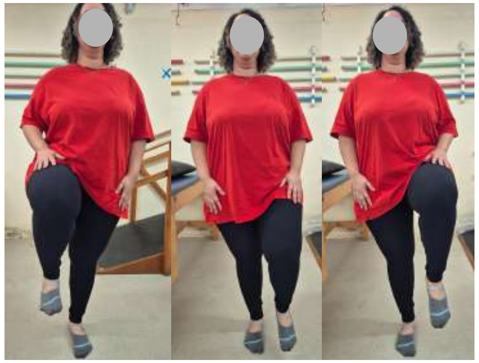

Trunk rotation

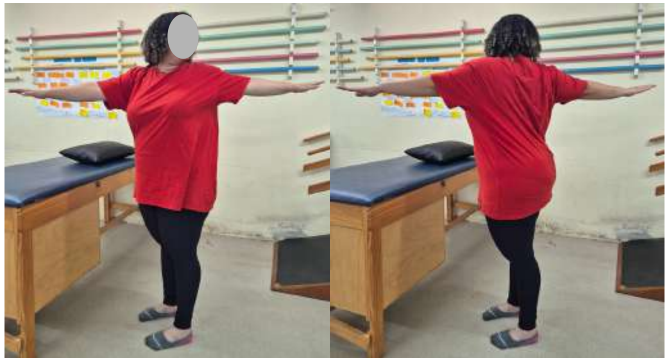

WARM-UP

Lower limb cycling

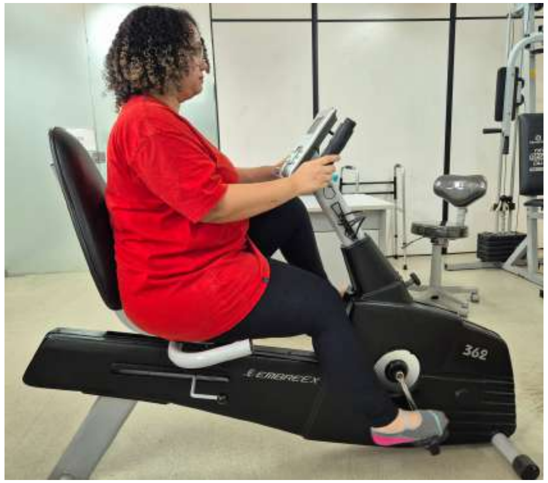

Upper limb cycling

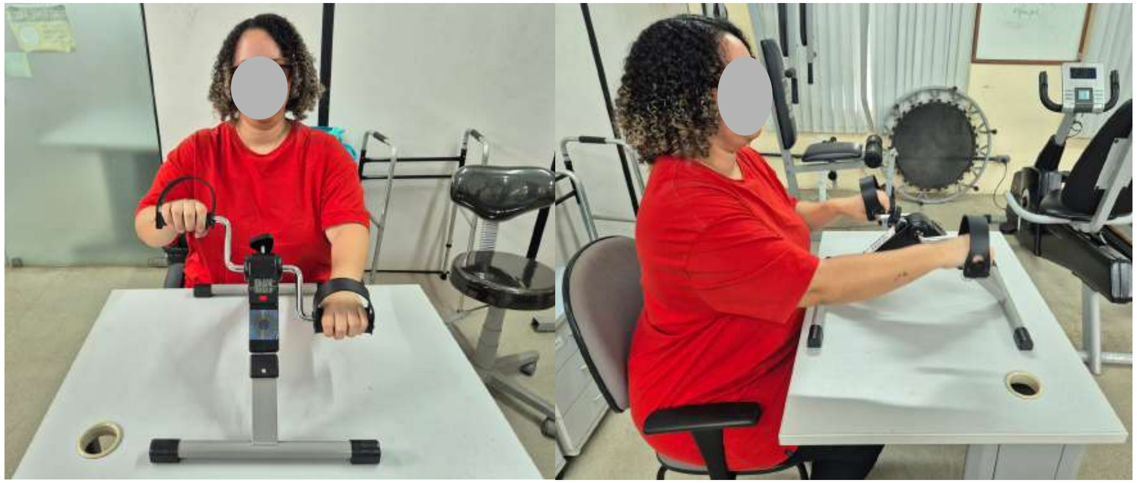

BRIDGE (PELVIC LIFT)

Week 1

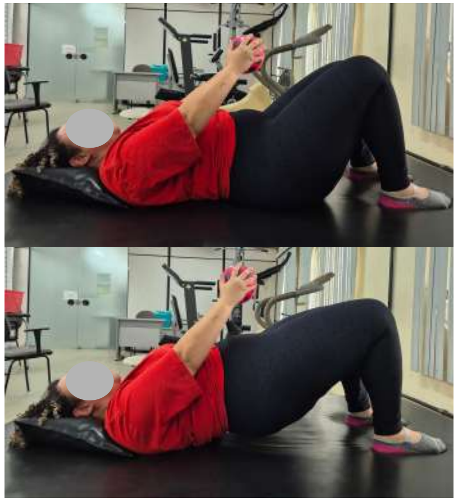

Weeks 2–4

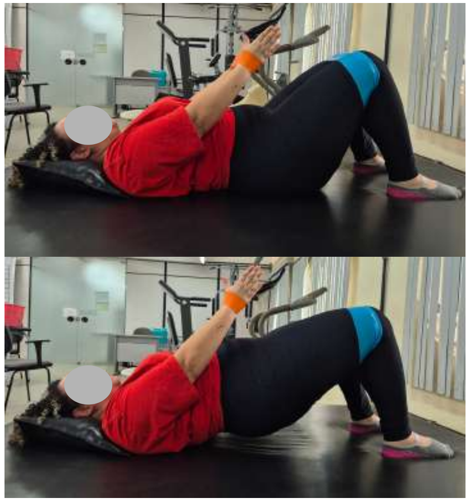

Weeks 5–7

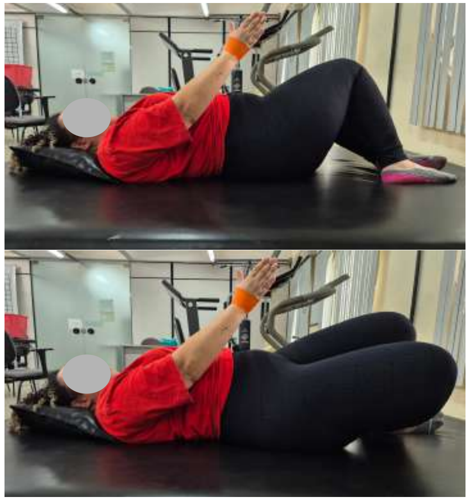

Weeks 8–10

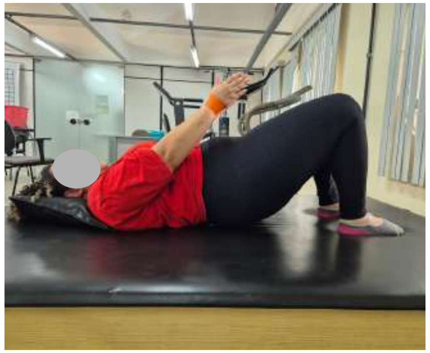

*isometric contraction halfway the movements

DIAGONAL TRUNK (DIAGONAL TRUNK FLEXION)

Week 1

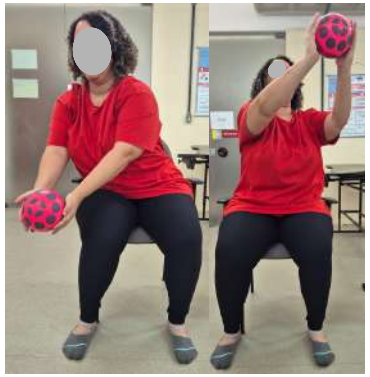

Weeks 2–4

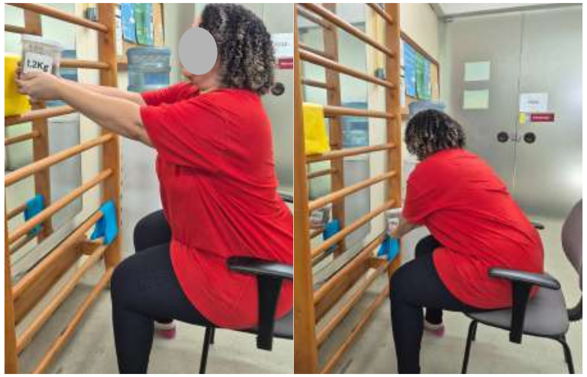

Weeks 5–7

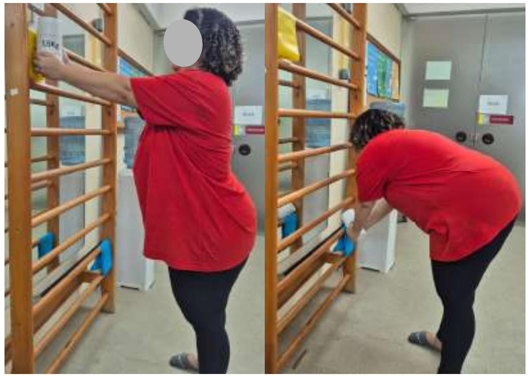

Weeks 8–10

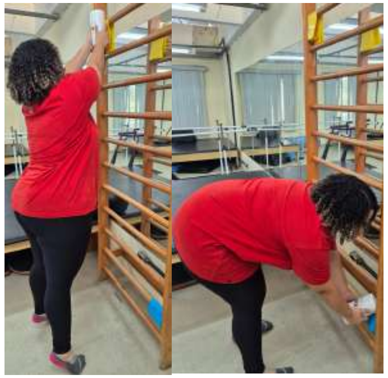

SIT-TO-STAND (SIT-TO-STAND FROM A CHAIR)

Week 1

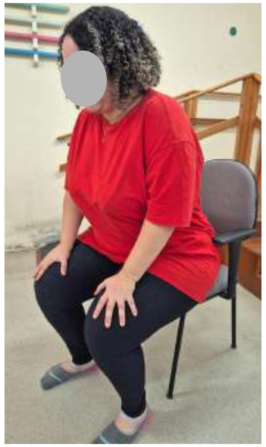

Weeks 2–4

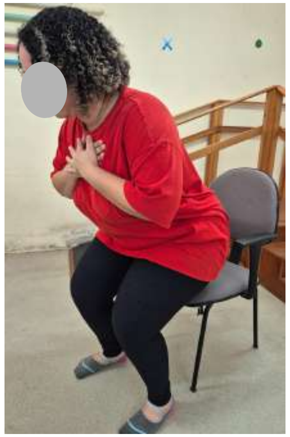

Weeks 5–7

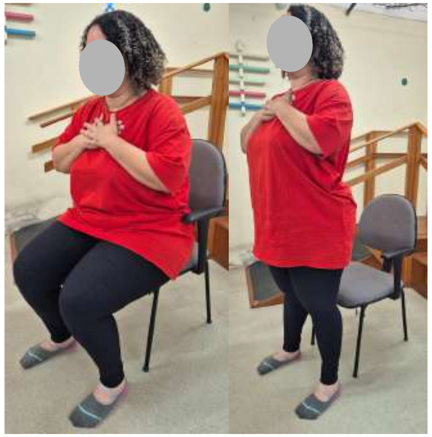

Weeks 8–10

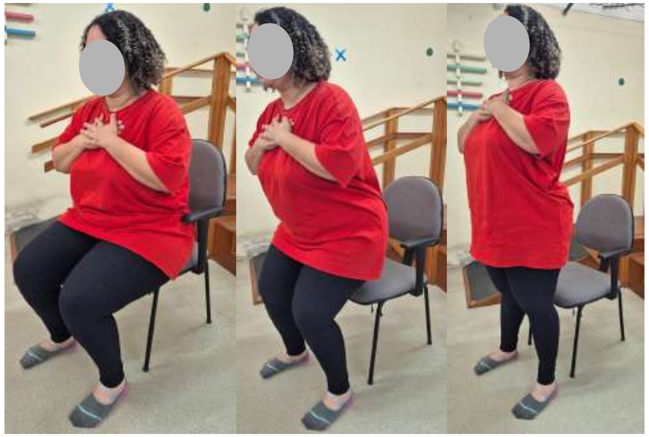

STEP TRAINING (STEP/ STAIR ASCENT AND DESCENT)

Week 1

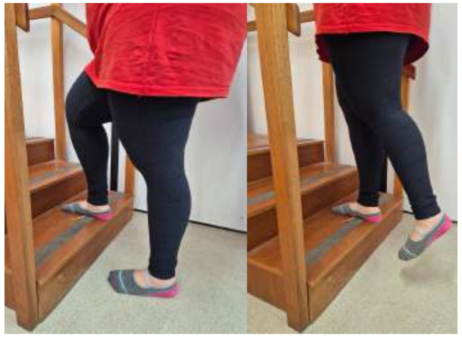

Weeks 2–4

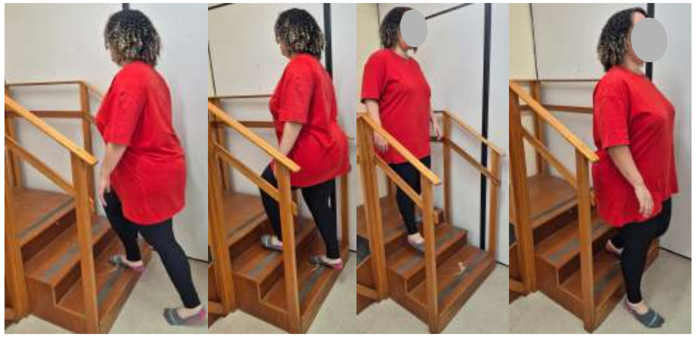

Weeks 5–7

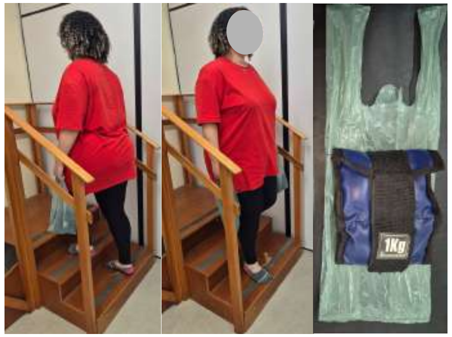

Weeks 8–10

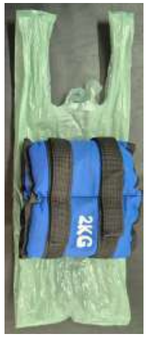

GAIT (GAIT CIRCUITS)

Week 1

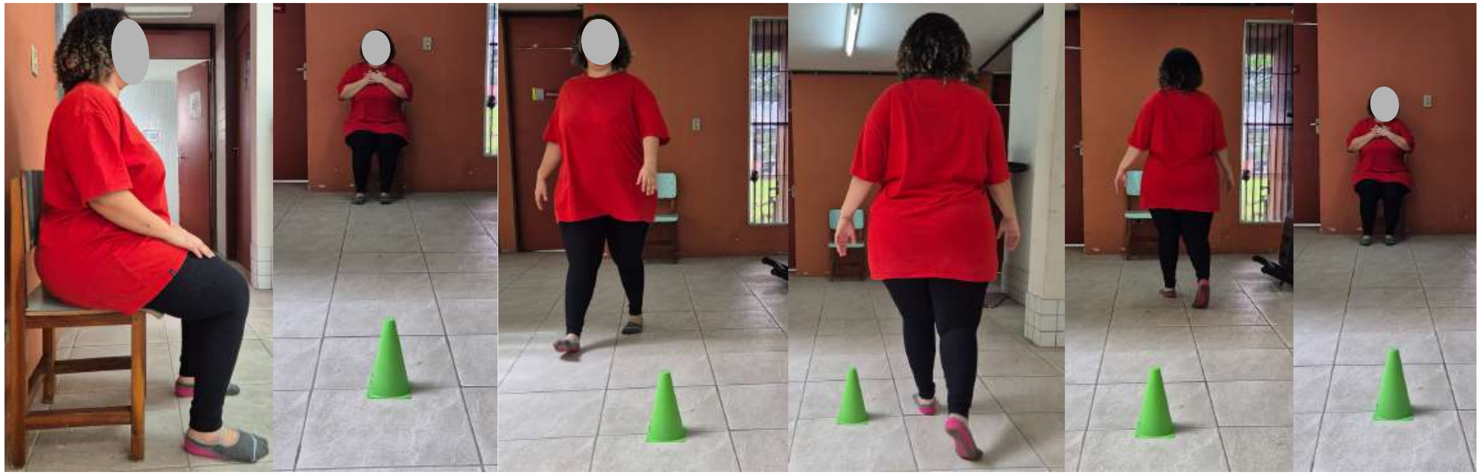

Weeks 2-4

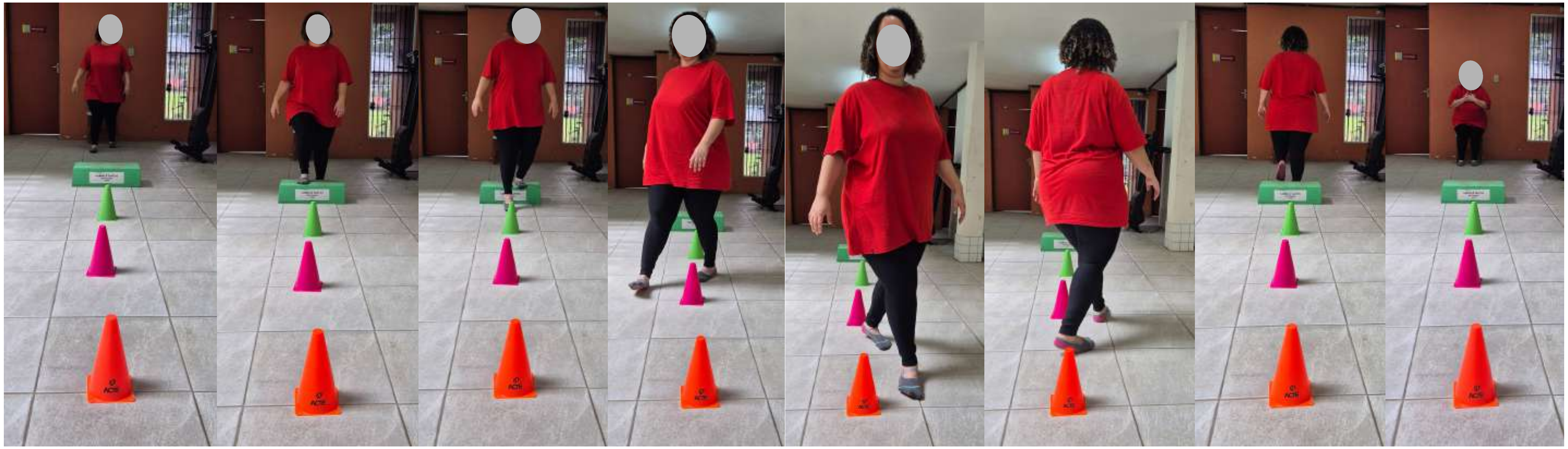

Weeks 5–7

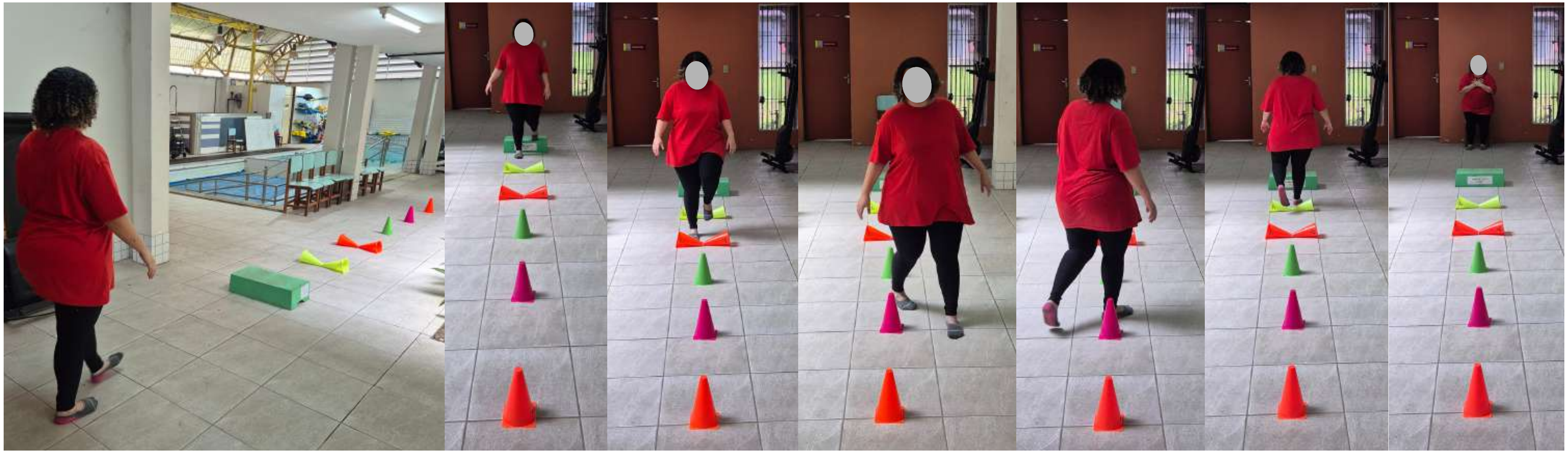

Weeks 8–10

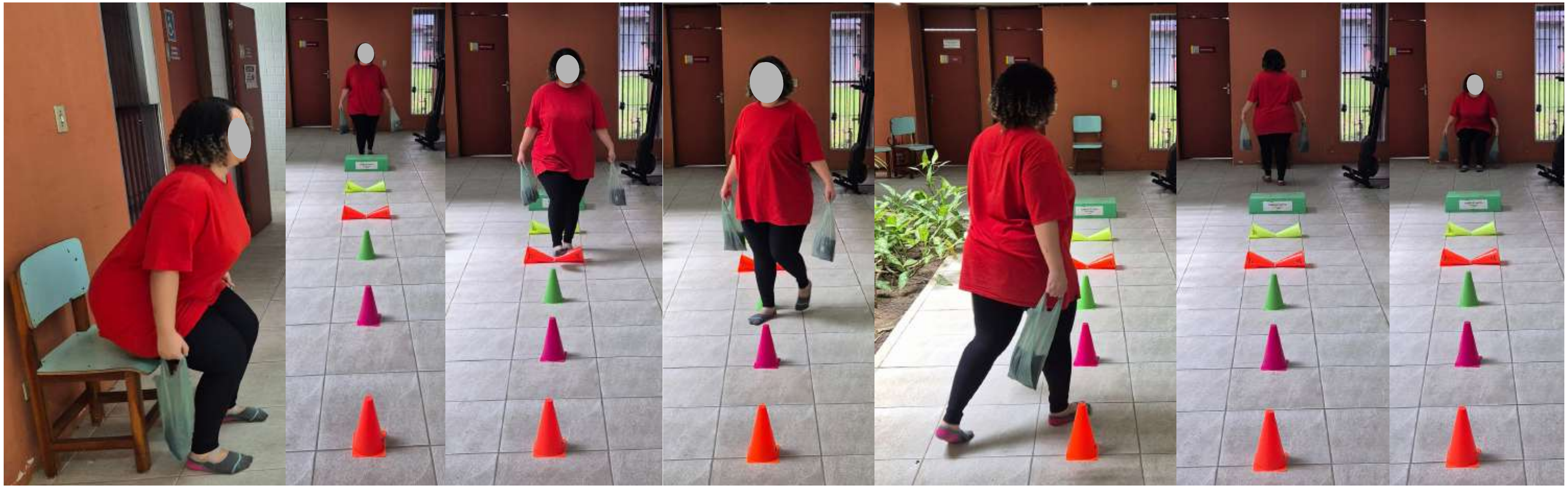

HAND EXERCISES (MOBILITY AND STRENGTHENING)

Transfer 10 varied pieces between containers

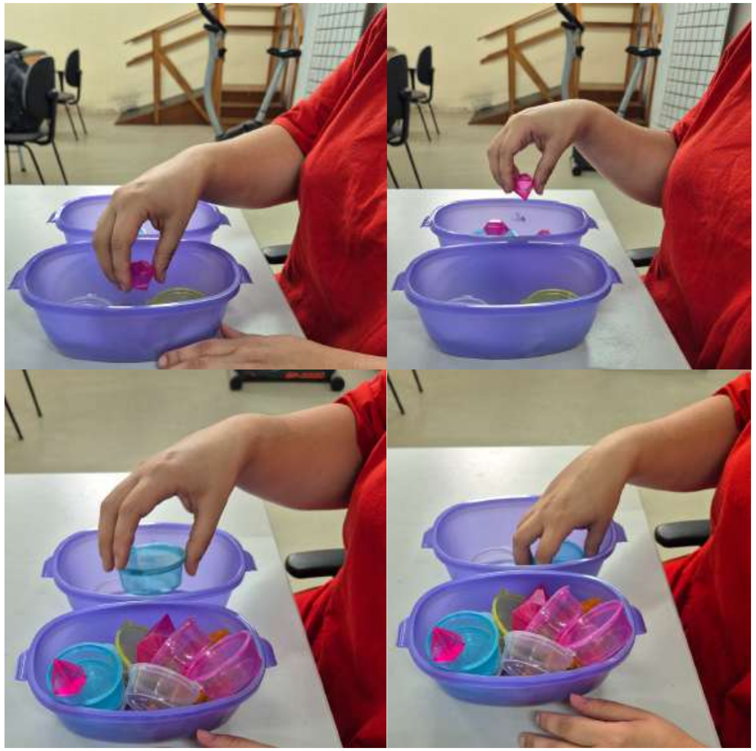

*over the weeks the number of repetitions and pieces increases

Roll/unroll cylindrical object

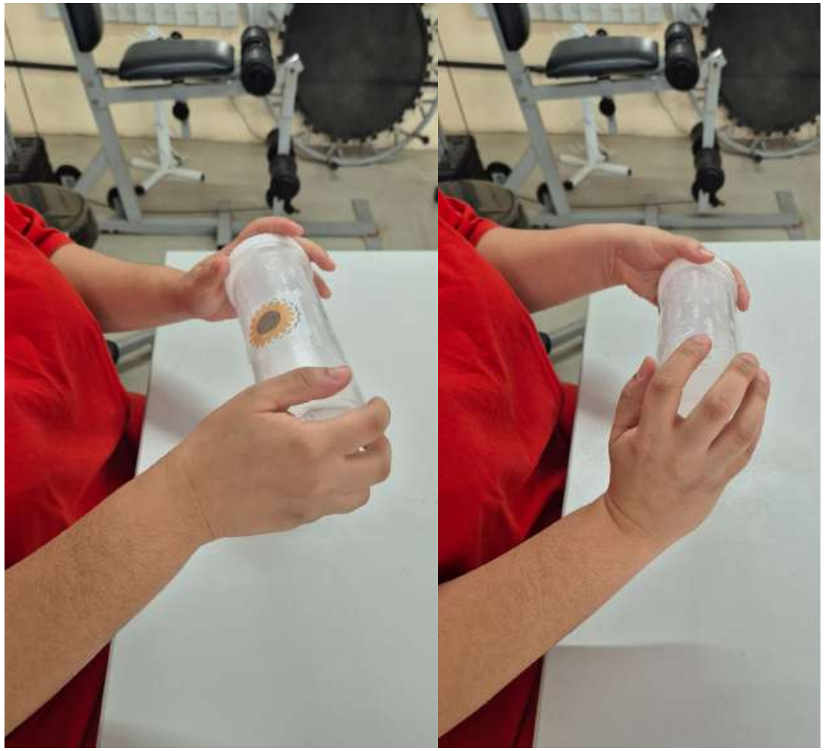

*over the weeks the number of repetitions increases

Open/close fingers against weak theraband

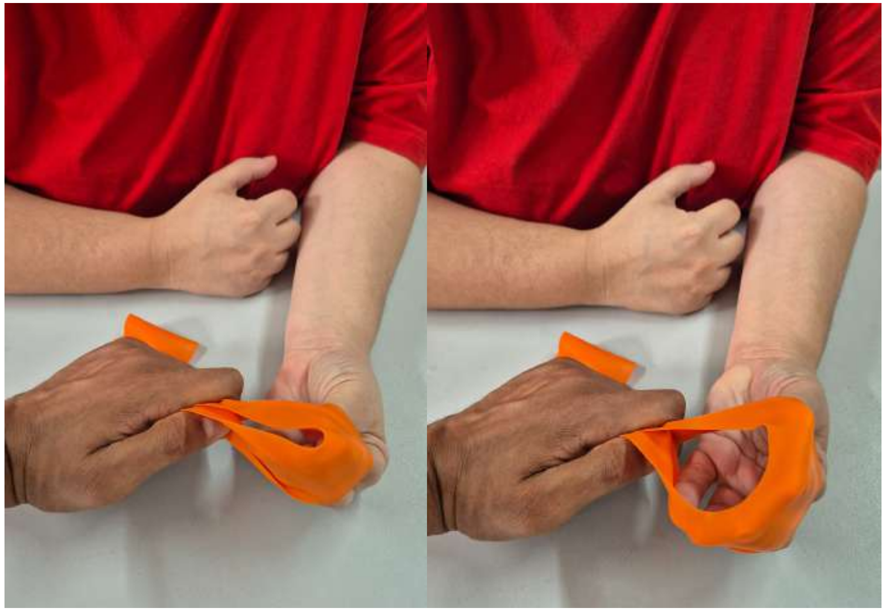

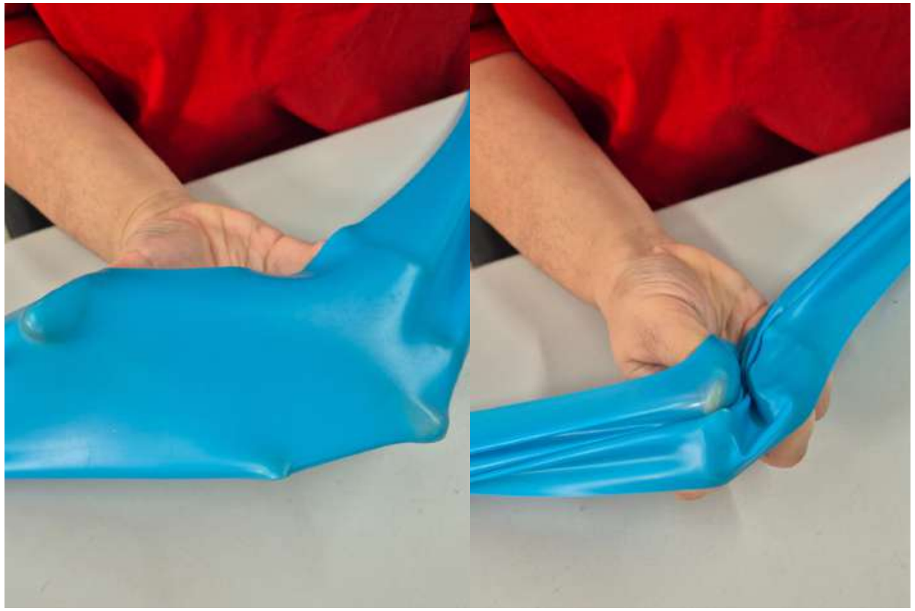

*over the weeks the number of repetitions and the resistance intensity increase
